# Supplementary material for: PLOS Computational Biology 2015 Reviewer Thank You
Source: PLoS Comput Biol. 2016 Feb 23;12(2):e1004815. doi: 10.1371/journal.pcbi.1004815 (PMC4764344; doi:10.1371/journal.pcbi.1004815)
Supplement: S1 Reviewer List — (PDF) [file pcbi.1004815.s001.pdf]

PLOS | COMPUTATIONAL BIOLOGY

PLOS Computational Biology would like to thank all those who reviewed on behalf of the journal in 2015:

Daniel Aalberts
Scott Aaronson
Henry Abarbanel
James Abbas
Craig Abbey
Hermann Aberle
Robert Abramovitch
Josep Abril
Luigi Acerbi
Orlando Acevedo
Christoph Adami
Frederick Adler
Boris Adryan
Tinri Aegerter-Wilmsen
Vera Afreixo
Ashutosh Agarwal
Ira Agrawal
Jacobo Aguirre
Alaa Ahmed
Hasan Ahmed
Natalie Ahn
Thomas Akam
Ilya Akberdin
Eyal Akiva
Sahar Akram
Tomas Alarcon
Larissa Albantakis
Reka Albert
Martí Aldea
Bree Aldridge
Helen Alexander
Alexander Alexeev
Leonidas Alexopoulos
Emil Alexov
Simon Alford
Samuel Alizon
Frederic Allain
Jun Allard
Carsten Allefeld
Benjamin Allen
Eivind Almaas
Jose-Manuel Alonso
Patrick Aloy
Jeff Alstott
Christian Althaus
Benjamin Althouse
Russ Altman
Eduardo Altmann
Philipp Altrock
Vikram Alva
Francisco Alvarez-Leefmans
Rommie Amaro
Ettore Ambrosini
Bagrat Amirikian
Uri Amit
Alexander Anderson
Noemi Andor
Isabelle Andre
R. David Andrew
Steven Andrews
Ioan Andricioaei
Ioannis Androulakis
Iris Antes
Maciek Antoniewicz
Haroon Anwar
Stefano Anzellotti
Miguel Aon
Lucy Aplin
Kevin Aquino
Leonardo Arbiza
Murat Arcak
Gil Ariel
Nimalan Arinaminpathy
Jeffrey Arle
Alain Arneodo
Markus Arnoldini
Mikko Arvas
Kenta Asahina
Fatemeh Ashari Ghomi
Randolph Ashton
Michael Assaf
Yassen Assenov
Collins Assisi
Craig Atencio
Canan Atilgan
Paul Atzberger
Benjamin Audit
Charles Auffray
Jean-Christophe Augustin
Robert Austin
Bruno Averbeck
Ferhat Ay
Nihat Ay
Francisco Azuaje
Marc Baaden
M. Madan Babu
Mohan Babu
Marco Bacci
Stephen Baccus
Omar Bagasra
Marc Baguelin
Timothy Bailey
Wyeth Bair
Chris Bakal
Joseph Bak-Coleman
Adam Baker
Douglas Bakkum
Gabor Balazsi
Nilesh Banavali
Rahul Banerjee
Edward Banigan
Martin Banks
Mukul Bansal
Shweta Bansal
Wolfgang Banzhaf
Lei Bao
Gyorgy Barabas
Omri Barak
Matteo Barberis
Virginia Barbour
Mary Barcellos-Hoff
Chris Barclay
Benjamin Bardiaux
Lee Bardwell
Izhar Bar-Gad
Naama Barkai
Christopher Barker
Stephanie Barnes
Lars Barquist

Perdita Barran
Alain Barrat
Ernest Barreto
Adam Barrett
Douglas Barrick
Endre Barta
Jennifer Bartell
Patrick Barth
Simon Barthelme
Ed Bartlett
Geoffrey Barton
Frederic Bartumeus
Chaitan Baru
Frederic Bastian
Ugo Bastolla
Effie Bastounis
Eric Batchelor
Alex Bateman
Mark Bathe
Alexis Battle
Davide Baù
Tuncay Baubec
Anaïs Baudot
Maxim Bazhenov
Jason Bazil
Jennifer Beane
Mark Beaumont
Timothy Beck
Oliver Beckstein
Attila Becskei
Peter Beemiller
Gerrit Beemster
Michael Beer
Niko Beerenwinkel
John Beggs
Farhad Behafarid
Ulrik Beierholm
Chase Beisel
George Bell
Martiniano Bello Ramirez
Joost Beltman
Pedro Beltrao
Jan Benda
Robert Bender
Alexandra Bendixen
Justin Benesch
Eshel Ben-Jacob
Matthew Bennett
Michael Bennett
Richard Benninger
Panayiotis Benos
Nir Ben-Tal
William Bentley
Andrew Berdahl
Omer Berenfeld
Philipp Berens
Johannes Berg
Bonnie Berger
Antony Beris
Ari Berkowitz
Francine Berman
Samuel Bernard
Simon Bernèche
Boris Bernhardt
Max Berniker
Andrew Bernoff
Stefano Berri
Hugues Berry
Guillaume Beslon
Robert Best
Michael Beyeler
Andreas Beyer
Hawthorne Beyer
Ilya Bezprozvanny
Upinder Bhalla
Sudin Bhattacharya
Guo-Qiang Bi
Jon Bielby
Philip Biggin
Patrice Bilesimo
Martin Billeter
Sebastian Binder
Ruthie Birger
Ewan Birney
David Biron
William Bishop
Marcia Bissaco
Kyle Bittinger
Rachael Blair
Judith Blake
Andrew Blanks
Danny Bluestein
Daniel Blustein
Nils Blüthgen
Kwabena Boahen
Nikolai Bode
Rainer Boeckmann
Norbert Boeddeker
Hinrich Boeger
Lies Boelen
Christophe Boete
Carl Boettiger
Rafal Bogacz
Gib Bogle
Konrad Böhm
Frederic Bois
Céline Boiteux
Dan Bolon
Peter Bond
Maciej Boni
Richard Bonneau
Alexandre M.J.J. Bonvin
Aarash Bordbar
Elhanan Borenstein
Alla Borisyuk
Peer Bork
Stefan Bornholdt
David Bortz
Guillaume Bourque
Teun Bousema
Alain Bousquet-Mélou
Isabelle Boutron
Ivana Bozic
Brooke Bozick
John Bradley
Tiago Branco
James Brandful
Kristina Branson
Siobhan Braybrook
Michelle Brazas
Michael Breakspear
Tinatin Brelidze
Björn Brembs
Andreas Bremges
Wieland Brendel
Michael Brent
Paul Bressloff
Romain Brette
Henry Brighton
Fiona Brinkman
Ryan Brinkman
Russell Brinkworth
Tom Britton
Tim Brodribb

Yana Bromberg
Charles Brook III
Mark Broom
Andre Brown
C. Titus Brown
Celeste Brown
Guy Brown
Sam Brown
Søren Brunak
Nicolas Brunel
Elizabeth Brunk
Mark Brynildsen
Vanni Bucci
Javier Buceta
Nicolae-Viorel Buchete
Matthias Buck
Caroline Buckee
Florian Buettner
Vincent Buffalo
Jerome Buhl
Michael Buhnerkempe
Laure Buhry
Martin Buist
Janusz Bujnicki
John Bukowy
Eric Bullinger
Dean Buonomano
Yoram Burak
Anthony Burkitt
Juan Burrone
Austin Burt
Zachary Burton
Tim Buschman
Harmen Bussemaker
Daniel Butts
Markus Butz
Helen Byrne
Chris Bystroff
Amedeo Caflisch
Patrick Cahan
David Cai
Tunahan Cakir
Isabelle Callebaut
Daniela Calvetti
Laurence Calzone
Carlo Camilloni
Kenneth Campbell
Matthew Campbell
Sharon Campbell
Stuart Campbell
Monica Campillos
Daniel Campos
Paulo Campos
Anne-Claude Camproux
Alberto Cañas
Carmen Canavier
Lulu Cao
Yang Cao
Jose Capitan
Emidio Capriotti
Luis Carillo
Johan Carlin
Anne Carlson
Bruce Carlson
David Carlson
Jonathan Carlson
Anders Carlsson
Shai Carmi
Carlos Carmona-Fontaine
Timothy Carpenter
Robert Carroll
Hannah Carter
Richard Carthew
Joao Carvalho
Rita Casadio
Gastone Castellani
Leandro Castellano
Mauro Castellarin
Romain Caze
Marco Cecchini
Gunnar Cedersund
Antonio Celani
Maurice Chacron
Monica Chagoyen
Benny Chain
Brian Chait
Jayprokas Chakrabarti
Hue Chan
Chuan-Hsiung Chang
Rui Chang
Dennis Chao
Claudine Chaouiya
Prem Chapagain
Mark Chaplain
John Chappell
Guillaume Charras
Steve Chase
Pratip Chattopadhyay
Pak-Lee Chau
Anuradha Chauhan
Luis Chaves
Tomas E. Cheatham
Gal Chechik
Alan Chen
Peng Chen
Rong Chen
Swaine Chen
Wei-Hua Chen
Xiang Chen
Xiaojie Chen
Xing Chen
Alan Cheng
Jianlin Cheng
Sen Cheng
Oleksandr Chepizhko
Cindy Chestek
Vincent C. K. Cheung
Dante Chialvo
Keng-Hwee Chiam
Roberto Chica
Lauren Childs
Hsuan-Chao Chiu
Pearl Chiu
Dong-Yeon Cho
Lillian Chong
Shantanu Chowdhury
Gerardo Chowell
Beat Christen
Jason Christie
John Christodoulou
Lon Chubiz
Rumi Chunara
Bong Jae Chung
Liang Chunguang
Radoslaw Cichy
Alexander Ciota
Giovanni Ciriello
Alessio Ciulli
Stanca Ciupe
Timothy Clark
Cathy Clarke
Richard Clayton
Cecilia Clementi
John Clithero

Claudia Clopath
Tyler Cluff
Murat Can Cobanoglu
Sarah Cobey
Luca Cocchi
Ruben Coen-Cagli
Michael Cohen
Murat Cokol
Carlo Colantuoni
Giles Colclough
Ryan Coleman
Caroline Colijn
Andrew Collins
Ivan Coluzza
Lucy Colwell
Luca Comai
Rory Conolly
Jessica Conway
Frank Coolen
Daniel Coombs
Mauro Copelli
Heather Cordell
Leighton Core
Bernat Corominas-Murtra
Manuel Corpas
Veronica Corrales Carvajal
Juan Cortes
Ivan Costa
Rui Costa
Susan Costantini
James Costello
Gary Cottrell
Steven Cox
Domenico Cozzetto
Karen Cranston
Trevor Creamer
Isaac Crespo
John Cressman
Ross Cressman
Frédéric Crevecoeur
Max Crispin
Deborah Cromer
Stephen Crotty
Holk Cruse
Miklos Csuros
Juan Cui
Qiang Cui
Qinghua Cui
Kathleen Cullen
Bruce Cumming
Hermann Cuntz
Christina Cuomo
Scott Currie
Gennady Cymbalyuk
András Czirók
Jack da Silva
Bernie Daigle
Neil Dalchau
Egidio D'Angelo
Hannelore Daniel
Michael Daniyan
Roy Dar
Jayajit Das
Moumita Das
Raibatak Das
Ranjan Dash
Harriet Dashnow
Susmita Datta
Jean Daunizeau
Stephen David
Jörn Davidsen
Lance Davidson
Stephen Davis
Adriana Dawes
Judy Day
Troy Day
Peter Dayan
Marcus de Aguiar
Daniela De Angelis
Gianni De Fabritiis
Vincent de Gardelle
Bert de Groot
Monique de Jager
Hidde de Jong
Xavier de la Cruz
Paolo De Los Rios
Nicola De Maio
Juan de Pablo
Gonzalo de Polavieja
Marcel de Reus
Dick de Ridder
Dirk de Ridder
Jeroen de Ridder
Bert De Rybel
David De Sancho
Erik De Schutter
Ive De Smet
Anita de Ward
Charlotte Deane
Greg DeAngelis
Simon Dedeo
Eric Deeds
Victor DeGruttola
Yves Dehouck
Thomas Deisboeck
Kirk Deitsch
Sara Del Valle
Domitilla Del Vecchio
Lucie Delemotte
Francesco Demayo
Francesca Demichelis
Robert Dempski
Wouter den Otter
Susan Denham
Matthew Denwood
Frantz Depaulis
Philippe Derreumaux
Maxime Descoteaux
Amit Deshwar
Christophe Dessimoz
Alain Destexhe
Andreas Deussen
Michael DeWeese
Chris DeZeeuw
Da Di
Diego Di Bernardo
Bendetto di Martino
Scott Diamond
Mederic Diard
Aaron Diaz
José Díaz
Casey Diekman
Markus Diesmann
Patrick Dillon
Frank DiMaio
Feng Ding
Jonathan Dingwell
Markus Dittrich
Narendra Dixit
Purushottam Dixit
Marko Djordjevic
Hana Dobrovolny
Rezarta Dogan
Takahiro Doi

Andrew Doig
Brent Doiron
Socrates Dokos
Michael Dolan
Eytan Domany
Carmen Domene
Bruce Donald
Alberto d'Onofrio
Denis Doorly
Pemra Doruker
Stéphane Douady
Rene Doursat
Linda Douw
Kenji Doya
Jeremy Draghi
John Drake
Brian Drawert
Patrick Drew
Ron Dror
Shaul Druckmann
Jan Drugowitsch
Huijing Du
Zhijun Duan
Renato Duarte
Eugene Duff
Ken Duffy
Michel Dumontier
Roland Dunbrack Jr.
Stanislaw Dunin-Horkawicz
Mary Dunlop
Geneviève Dupont
Salvador Dura-Bernal
Miquel Duran-Frigola
Volker Dürr
Julien Dutheil
Daniel Dwyer
Rosemary Dyson
Peter Eastman
Martin Ebeling
Alexander Ecker
Miguel Eckstein
Andrew Edwards
Felix Effenberger
Ulrich Egert
Robert Egger
Stephen Eglen
Oliv Eidam
Thorsten Eierhoff
Gaute Einevoll
Wolfgang Einhäuser
Thomas Eiting
Orjan Ekeberg
Meriem El Karoui
Avigdor Eldar
Seif Eldawlatly
James Elder
Coen Elemans
Olivier Elemento
Donald Elmore
Arne Elofsson
Yasmine El-Shamayleh
Timothy Elston
Thierry Emonet
Heiko Enderling
Jan Engelstädter
Bernhard Englitz
Emilia Entcheva
Ozlem Equils
Fabian Erdel
A. Murat Eren
Amir Erez
Udo Ernst
Thomas Erren
Jesús Espinal-Enríquez
Carlos Espinosa-Soto
Stephan Ewert
Eduardo Eyras
Adam Eyre-Walker
Donald Faber
James Faeder
Adrienne Fairhall
Martin Falcke
Bjoern Falkenburger
Daniel Falush
Xiaodan Fan
Christopher Fang-Yen
Etienne Farcot
Damien Farine
Matteo Farinella
Yossi Farjoun
Illés Farkas
Brian Fath
Segun Fatumo
Karoline Faust
Vadim V. Fedorov
Ariel Feiglin
Ofer Feinerman
Adam Feist
Ten Feizi
Marcus Feldman
James Feng
Juan Fernandez-Recio
Matthew Ferrari
Ila Fiete
Marc Figge
Grazziela Figueredo
James Finley
Stacey Finley
Hilary Finucane
Andrew Fiore-Gartland
Brian Fischer
Andras Fiser
Jeffrey Fisher
Patrick Flaherty
Sarel Fleishman
Roland Fleming
Stephen Fleming
Alexander Fletcher
Michael Flossdorf
Anthony Fodor
Aaron Fogelson
Ariele Follis
Stephen Fong
Christopher Fonnesbeck
Bertrand Fontaine
Daniel Forger
Julie Forman-Kay
David Foster
Douglas Fowler
Philip Fowler
Seth Fraden
Paul Francois
Marcos Frank
Michael Frank
David Franklin
Eric Franzosa
Christophe Fraser
James Fraser
Peter Fraser
Jeffrey Fredberg
Benjamin J. Fregly
Clark Freifeld
Alfredo Freites
Atle Fretheim

Leonid Fridlyand
Hermann Frieboes
Jonathan Friedman
Benjamin Friedrich
Karl Friston
Robert Froemke
Holger Fröhlich
Simon Frost
Feng Fu
Yan Fu
Julian Fuchs
Tomoki Fukai
Vincent Funari
Sebastian Funk
Cristina Furdui
Terrence Furey
Nicholas Furl
Nicholas Furnham
Chikara Furusawa
Stefano Fusi
Terry Gaasterland
Fabrizio Gabbiani
Roberto Galán
Giovanni Galizia
Alvaro Galli
Emilio Gallicchio
Adriana Galvan
Oxana Galzitskaya
Manoj Gambhir
Nikita Gamper
Vitaly Ganusov
Xin Gao
Vytautas Gapsys
Angel Garcia
Thomas Garcia
Victor Garcia
Jose Manuel Garcia Aznar
Jordi Garcia-Ojalvo
Melissa Gardner
Timothy Gardner
Jennifer Gardy
Yuval Garini
Lana Garmire
Susan Gasser
Michael Gastner
Robert Gatenby
Tom Gaunt
Katharina Gaus
Daniel Gautheret
Hao Ge
Christophe Geldmacher
Ignacio General
Guy Genin
Robert Gentleman
Andrew Gentles
Guy Georges
Mark Georgeson
George Georgiou
Apostolos Georgopoulos
Philip Gerlee
Samuel Gershman
Wulfram Gerstner
David Gfeller
Dario Ghersi
Geoffrey Ghose
Kingshuk Ghosh
Samik Ghosh
Amel Ghouila
Stephano Gianni
Sean Gibbons
David Gifford
Frank Gijsen
Luis Gilarranz
Giorgio Gilestro
Matthieu Gilson
Jeffrey Gimble
Anne-Claude Gingras
Lisa Giocomo
Jesus Giraldo
Mark Girolami
Anthony Gitter
Aryn Gittis
Michele Giugliano
Alessandro Giuliani
Chad Giusti
Erida Gjini
Julijana Gjorgjieva
Margaret Glasner
Jan Glatz
Adam Godzik
Pranay Goel
Lea Goentoro
Adi Goldenzweig
Joshua Goldwyn
Tim Gollisch
David Golomb
Mark Gomelsky
Shawn Gomez
Jorge Gomez Tejeda Zanudo
Beatriz Gomis
Mehmet Gönen
Joaquin Goni
Keith Gooch
Benjamin Good
Geoffrey Goodhill
Uri Gophna
Raluca Gordan
Deborah Gordon
Jeff Gore
Alemayehu Gorfe
Monica Gori
Shachi Gosavi
Graeme Gossel
Katalin Gothard
Susumu Goto
Cara Gottardi
Raphael Gottardo
Raphael Gottardo
Alexandros Goulas
Nir Gov
Igor Goychuk
Manfred Grabherr
Paul Graham
Trevor Graham
Alexandre Gramfort
Eleonora Grandi
Francois Graner
Pascal Grange
Christine Granier
Barry Grant
Henk Granzier
Frauke Gräter
Michael Graupner
Simon Gravel
Julien Gravier
Frederik Graw
Larry Gray
C. Shawn Green
David Greenberg
Casey Greene
Christopher Greenman
Joseph Greenstein
T. Ryan Gregory
Emmanuel Grenier

Martin Greschner
Paul Gribble
Paolo Grigolini
Gevorg Grigoryan
Jacopo Grilli
Ramon Grima
Nick Grishin
Iris Groen
Gerrit Groenhof
M. Michael Gromiha
Ilan Gronau
Richard K. Grosberg
Claudio Grosman
Thilo Gross
Alan Grossfield
Sonja Gruen
Jan Gründemann
Dmytro Grytskyy
Liqun (Andrew) Gu
Yongtao Guan
Yuanfang Guan
John Guatelli
Burak Güçlü
Jeremie Guedj
Jeremie Guedj
Frédéric Guichard
Roderic Guigo
James Gumbart
Rudiyanto Gunawan
Aysegul Gunduz
Kris Gunsalus
Chao-Yu Guo
Daqing Guo
Guangwu Guo
Todd Gureckis
Dan Gusfield
Martin Guthold
Robert Gütig
Olgun Guvench
John Hackett
Oliver Hädicke
Ralf Haefner
Dieter Haemmerich
Hiroshi Haeno
Jan Haerter
Tzachi Hagai
Michael Hagan
Stephen Hagen
Matthew Hahn
Steve Hahn
Adrian Haith
Marc Halfon
Turkan Haliloglu
Oskar Hallatschek
Bjarni Halldórsson
Heiko Hamann
Olivier Hamant
Donald Hamelberg
James Hamilton
Xiaonan Han
William Hanage
Andreas Handel
Jacob Hanna
David Hansel
Elsa Hansen
Jens Hansen
Fan Hao
William Harcombe
Todd Hare
John Hargrove
Matthew Harrison
H.C. Harsha
G. Traver Hart
Gal Haspel
Nurit Haspel
Dimitris Hatziavramidis
Vassily Hatzimanikatis
Bernard Haubold
Helmut Hauser
Peter Haverty
Guy Hawkins
Ian S. Haworth
Boris Hayete
John-Dylan Haynes
Vincent Hayward
Daihai He
Xin He
Dieter Heermann
Nicolas Heess
Ines Heiland
Elmar Heinzle
Jakob Heinzle
Leonhard Held
Marc Hellerstein
Per Hellstrand
Brian Helmke
Volkhard Helms
Martin Hemberg
Charlotte Hemelrijk
Diana Hendrickx
Craig Henriquez
Christopher Henry
Nelly Henry
Roslyn Henry
Niel Hens
Burkhard Hense
Joshua Herbeck
Rigoberto Hernandez
Miguel Herrero
Markus Herrgard
Uri Hershberg
John Hertz
Hanspeter Herzel
David Herzfeld
Ben Hescott
Stephanie Hicks
Alicia Hidalgo
Winston Hide
Andrew Higginson
Christian Hilbe
Falk Hildebrand
Ronald Hills
Jane Hillston
Daniel Himmelstein
Anjali Hinch
Tomoo Hirano
Yoshito Hirata
William Hlavacek
Katherine Hoadley
Birte Höcker
Toby Hocking
Philip Hodgkin
Gabriel Hoffman
Kay Hofman
Martin Hofmann-Apitius
Matan Hofree
David Holcman
Arun Holden
Benjamin Holder
Guillaume Hollard
Edward Hollox
Petter Holme
Jeffrey Holmes
Jens Holst

Anthony Holtmaat
Hermann-Georg Holzhütter
Hisao Honda
Ha Hong
Sungho Hong
Barry Honig
Raquel Hontecillas
Stefan Hoops
Thomas Hopf
Frank Hoppensteadt
Dominik Horinek
Amnon Horovitz
John Houde
Thomas House
Martin Howard
Peter Hraber
Li Hsu
Yin Hu
Kerwyn Huang
Xiaolei Huang
Xuhui Huang
Florence Hubert
Ramon Huerta
David Hughes
Nicholas Hughes
Sean Humbert
James Humble
Gerhard Hummer
Mark Humphries
Thomas Hund
C. Anthony Hunt
Daniel Huson
Mikael Huss
Cendri Hutcherson
Alexander Huth
Curtis Huttenhower
Eun Jung Hwang
Wonmuk Hwang
Alexandre Hyafil
Changbong Hyeon
Lilia Iakoucheva
Marta Ibañes
Oleg Igoshin
Ioannis Iliopoulos
Chris Illingworth
Gianluca Interlandi
Christos Ioannou
Ed Ionides
Ivan Iossifov
Junji Ito
Robert Ivanek
Ivaylo Ivanov
Shingo Iwami
Jun Izawa
Leighton T. Izu
Andrew Jackson
Donald Jacobs
Robert Jacobs
Matthew Jacobson
Vincent Jacquemet
Dieter Jaeger
Saleet Jafri
Harsh Jain
Jozsi Jalics
Abdul Salem Jarrah
Constance J. Jeffery
Anil Jegga
Danyel Jennen
Lars Jensen
Mark Jensen
Mogens Jensen
Ole Jensen
Paul Jensen
Yong Hyun Jeon
Robert Jernigan
Shuiwang Ji
Peilin Jia
Hui Jiang
Jack Jiang
Yi Jiang
Alexandra Jilkine
Dezhe Jin
Yong-Su Jin
Viktor Jirsa
Mats Jirstrand
Jenia Jitsev
Paul Johnson
Philip Johnson
Wilsaan Joiner
Vladimir Jojic
Gary Jones
Henrik Jonsson
Rebecka Jornsten
Robert Josephs
Sanjay Joshi
Daniel Jost
Jurgen Jost
Goo Jun
Suckjoon Jun
Ivan Junier
Petr Jurecka
Davor Jureti C
Joe Kable
Lars Kaderali
James Kadonaga
Mads Kærn
Ran Kafri
Marcus Kaiser
Marko Kaksonen
Christoph Kaleta
Olga Kalinina
Lukas Käll
Charalampos Kalodimos
Atanas Kamburov
Yukiyasu Kamitani
Bjoern Kampa
Ryota Kanai
Hyun Min Kang
Shuli Kang
Ingmar Kanitscheider
Maricel Kann
Natarajan Kannan
Jitendra Kanodia
Nancy Kanwisher
Rowland Kao
Noam Kaplan
Tommy Kaplan
John Karanicolas
George Karniadakis
Jonathan Karr
Matthias Kaschube
David Kashatus
Makio Kashino
Panagiotis Kastritis
William Kath
Koichi Kato
Saul Kato
Marceille Kaufman
Ibrahim Kavakli
Kamran Kaveh
Mitsunori Kayano
Hokto Kazama
Yiannis Kaznessis
Dukka Kc

Ruian Ke
Benjamin Kear
Amy Keating
Matt Keeling
Bettina Keller
Laurent Keller
Miklos Kellermayer
Manolis Kellis
Douglas Kellogg
Eric Kelsic
David Kelvin
Melissa Kemp
Eben Kenah
Richard Kennaway
David Kennedy
Rex Kerr
Sinan Keten
Syma Khalid
Mehdi Khamassi
Ankit Khambhati
Bhavin Khatri
Natalia Khuri
Jeff Kidd
Stefan Kiebel
Scott Kight
Daisuke Kihara
Yasuyuki Kihara
Peter Killeen
A. Marm Kilpatrick
Zachary Kilpatrick
Eunjung Kim
Junhyong Kim
Philip Kim
Seyoung Kim
Sun Kim
Taeyoon Kim
Yoo-Ah Kim
Marek Kimmel
Ian Kinchin
Aaron King
Andrew King
Zachary Andrew King
Osame Kinouchi
Steffen Klamt
Jeffery Klauda
Frederick Klauschen
Max Kleiman-Weiner
Ulrich Kleinekathoefer
Thorsten Klingen
David Klinke
Don Klinkenberg
Edda Klipp
Andreas Kloetgen
Yuval Kluger
Bernhard Knapp
David Knapp
Mark A. Knepper
Michael Knop
Dmitry Kobak
Jaroslav Koca
Jacob Koella
Rainer Koenig
Roger Koeppe
Heinz Koeppl
John Koland
Richard Kollar
Nils Kolling
Natalia Komarova
Tamiki Komatsuzaki
Elizabeth Komives
Jane Kondev
Genevieve Konopka
Anna Konstor
Oliver Korb
Philipp Korber
Tamás Korcsmáros
Alexandr Kornev
Kirill Korolev
Dean Korosak
Tanja Kortemme
Sergei Kosakovsky Pond
Klemen Koselj
Andrej Kosmrlj
Roger Kouyos
Ozan Koyluoglu
Mehmet Koyuturk
Dima Kozakov
Michael Kozlov
Robert Kozma
Eric Kramer
Mark Kramer
Robert Krams
David Kreil
Andreas Kremling
Thomas Kreuz
Moritz Kreysing
Skirmantas Kriaucionis
Oleg Krichevsky
Birgit Kriener
Morten Kringelbach
Sandeep Krishna
J. Krishnan
Sergey Kryazhimskiy
Maik Kschischo
Jan Kubelka
Jason Kubinak
Roman Kuc
Adam Kucharski
Andrzej Kudlicki
Brian Kuhlman
Leslie Kuhn
Aloke Kumar
Arvind Kumar
Arthur Kuo
Ozge Kurkcuoglu
Daisuke Kuroda
Shinya Kuroda
Vartan Kurtcuoglu
Zeb Kurth-Nelson
Edo Kussell
Jason Kutch
Serdar Kuyucak
Taejoon Kwon
Alexey Ladokhin
Benoit Ladoux
Alain Laederach
Kevin Lafferty
Jens Lagergren
Nicola Lai
Elodie Laine
Alessandro Laio
Nan Laird
Melike Lakadamyali
Jeremy Lakey
Ashish Lal
Tommy Lam
Tracey Lamb
Arthur Lander
Kerry Landman
Christian Landry
David Landsman
Michael Landy
Jeffry Lansman
Anders Lansner

Jeroen Laros
Daniel Larremore
Nicholas Larson
Michael Lassig
Peter Latham
Kenneth Latimer
Matthew Lau
Mark Laubach
Kenneth Laurita
Martin Lauss
Conor Lawless
Michael Lawrence
Daniel Laydon
Anita Layton
Matthew Lazzara
Arnaud Le Menach
Mikhail Lebedev
Julie Lecomte
Kevin Leder
Alan Lee
Michael Lee
Phil Lee
Sangwan Lee
Su-In Lee
Uncheol Lee
Rob Leech
Chae Hun Leem
Marie-Paule Lefranc
Klaus Lehnertz
Joel Leibo
Christian Leibold
Chris-Andre Leimeister
Tanya Leise
Thomas Leitner
Patrick Lemaire
Louis Lemieux
Edward Lemke
Justin Lemkul
Marc Lenburg
Boris Lenhard
Marc Lensink
Anthony Leonardo
Michael Lerner
Justin Lessler
Gabriel Leventhal
Anna Levina
Erel Levine
Michael Levine
Sivan Leviyang
Emmanuel Levy
Yaakov Levy
Daniel Lew
Nathan Lewis
Fran Lewitter
Joel Lexchin
Jingjing Li
Jun Li
Lang Li
Lei Li
Liwu Li
Tong Li
Wenyuan Li
Xiaoli Li
Yue Li
Zheng Li
Han Liang
Hualou Liang
Jie Liang
Po-Huang Liang
Richard Liang
Chung-Min Liao
James Liao
Shuohao Liao
Eric Libby
David Liberles
Igor Libourel
Olivier Lichtarge
Tami Lieberman
Sara Light
Markus Lill
Vittorio Limongelli
Kevin Lin
Yu-Shan Lin
Maurine Linder
Stinus Lindgreen
Rune Linding
Tom Lindström
Doro Lindtke
Glenn Lines
Matthew Links
Marja-Leena Linne
Christiane Linster
Sébastien Lion
Tomasz Lipniacki
Christoph Lippert
Stefano Lise
Polina Lishko
Jacob Litman
Ashok Litwin-Kumar
Jian Liu
Peng Liu
Weidong Liu
Yang-Yu Liu
Dennis Livesay
Adi Livnat
James Lloyd-Smith
Alexander Lobkovsky
Daniel Lobo
John Lock
James Locke
Shawn Lockery
Eric Londin
Liam Longo
Pedro Lopes
Lester Loschky
Matthieu Louis
Madeleine Lowery
Ju Lu
Ting Lu
Zhiyong Lu
Zhong-Lin Lu
Vassiliy Lubchenko
David Lubensky
Sharon Lubkin
Christopher Lucas
Jörg Lücke
Elliot Ludvig
Casimir Ludwig
E. Georg Luebeck
Benedict Luensmann
Desmond Lun
Jingqin Luo
Ruibang Luo
Andrei Lupas
Cheng Ly
Cliff Lynch
Penelope Lynch
Shina Caroline Lynn Kamerlin
Grant Lythe
Katrina Lythgoe
William Lytton
Buyong Ma
Lan Ma
Wenzhe Ma

Wolfgang Maass
Feilim Mac Gabhann
Ben MacArthur
Stuart MacGregor
Philip Machanick
Geoff Macintyre
Jakob Macke
Alexander MacKerell
Carol MacKintosh
Paul Macklin
Adam MacLean
Jason MacLean
Malcolm Macleod
Shev MacNamara
Daniel MacNulty
Greg Madej
Jeffry Madura
Carsten Magnus
Gesham Magombedze
Radhakrishnan Mahadevan
Joe Mahaffy
Adam Mahdi
Louis Maher III
Ari Pekka Mahonen
Shaun Mahony
Manuel Mai
John Maina
Leonard Maler
Wasim Malik
Laurence Maloney
Pascal Mamassian
Ilgar Mamedov
Piero Manfredi
Michael Mangold
P. Manimaran
M. Manivannan
Tiina Manninen
Michael Manolidis
Ohad Manor
Jesus Manrique
Michael Manson
Sayed-Amir Marashi
Daniel Marbach
Adam Marblestone
Paolo Marcatili
Daniele Marinazzo
Nikola Markov
Thomas Marlovits
Shimon Marom
Tatiana Marquez-Lago
Olivier Marre
Gary Marsat
Tobias Marschall
Alison Marsden
Joseph Marsh
James Marshall
Marie-Claude Marsolier-Kergoat
Lennart Martens
Brent Martin
Iñigo Martincorena
Luis Martinez
Marc Marti-Renom
Georg Martius
Maryann Martone
Yosef Maruvka
Christopher Marx
Alex Marzel
George Mashour
Sergei Maslov
Nicolas Masse
Naoki Masuda
William Mather
Tom Matheson
Alexander Mathis
Frederick Matsen IV
Andrea Mattevi
Louis Matthieu
Victor Matveev
Olga Matveeva
Michael Mauk
Gerrit Maus
Philip Maybank
Bruce Mayer
Fiona McCarthy
James McCaw
Megan McClean
Sam Mcclure
Andrew McDavid
Jason McDermott
Mark McDonnell
Alison McGuigan
Joseph McGuire
A. Randy McIntosh
David McIver
David McMillen
Paul McMurdie
James Meadow
Pieter Medendorp
Timothy Meese
Ahmed Mehdi
Mehrdad Mehrbod
Pankaj Mehta
Herbert Meiselman
Francisco Melo
Manuel Melo
Raoul-Martin Memmesheimer
Eduardo Mendoza
Yilin Meng
Vinod Menon
Daniele Merico
Roeland Merks
Maarten Merkx
Arnaud Messé
Jordi Mestres
Sarah Metrustry
Markus Meuwly
Austin Meyer
Martin Meyer
Florent Meyniel
George Mias
Magali Michaut
Andrea Micheletti
Cristian Micheletti
Stephen Michnick
Richard (Rick) Michod
Nicole Mideo
Luis Mier-Y-Teran-Romero
Daniel Mietchen
Michele Migliore
Tijana Milenkovic
Manfred Milinski
Christopher Miller
Paul Miller
Ryan Mills
Peter Minary
Gary Mirams
Tom Misteli
Christopher Mitchell
John Mitchell
Katie Mitchell-Koch
Tanja Mittag
Vivek Modi

Mohammad Mofrad
Alex Mogilner
Debasisa Mohanty
Samat Moldakarimov
David Molony
Singh Mona
Remi Monasson
M. Anthony Moody
Tae Seok Moon
Brian Moore
James Moore
Paul Moore
Thierry Mora
Mahmoud Moradi
Rosalyn Moran
Faruck Morcos
Yves Moreau
Luis Morelli
Ed Moreno
Ruben Moreno-Bote
Dimitrios Morikis
Takako Morimoto
Shinichi Morishita
Richard Morton
Roberto Mosca
Mathieu Moslonka-Lefebvre
Thiago Mosqueiro
Sara Mostafavi
Rafal Mostowy
Sebastien Motsch
Adilson E. Motter
Andrea Mozzarelli
Florian Mueller
Andrew Mugler
Eran Mukamel
Sayan Mukherjee
Sarah Muldoon
Bertram Müller-Myhsok
Steven Munger
Lance Munn
Brian Munsky
Marieke Mur
Katsuhiko Murakami
Virgil Muresan
Robert Murphy
John Murray
Richard Murray
Ben Murrell
Mark Musen
Ferdinando Mussa-Ivaldi
Ville Mustonen
Michael Mwangi
Chris Myers
Simon Myers
Jean-Pierre Nadal
Carey Nadell
Elena Nadezhdina
Suhita Nadkarni
Swati Nagar
Valentin Nagerl
Rafael Najmanovich
Kae Nakamura
Masanori Nakamura
Luay Nakhleh
Atsushi Nambu
Vagheesh Narasimhan
Rishikesh Narayanan
Marko Nardini
Jatin Narula
Gerard Nash
Richard Naud
Jeremie Naude
Ali Navid
Martin Nawrot
Daniel Neafsey
Sriram Neelamegham
Emre Neftci
Richard Neher
Stuart Neil
Ali Neishabouri
Martin Nelson
Ilya Nemenman
Theoden Netoff
Gregor Neuert
Avidan Neumann
Heiko Neumann
Anje-Margriet Neutel
Aurelie Neveol
Susana Neves
Stuart Newman
Van Ngo
Charles Nicholson
Stamatios Nicolis
Philip Nicovich
Qing Nie
Steven Niederer
Jens Nielsen
Lars Nielsen
Vipavee Niemsiri
Sebastian Nijman
Michael Nikolaidis
Zoran Nikoloski
Vadim Nikulin
Michael Nilges
Lennart Nilsson
Roland Nilsson
Hafumi Nishi
Masha Niv
Robert Noble
Frank Noé
Katharina Nöh
Guido Nolte
Intawat Nookaew
Javad Noorbakhsh
Magnus Nordborg
Akinao Nose
Sergei Noskov
Richard Notebaart
Armita Nourmohammad
Houtan Noushmehr
Thomas Nowotny
Daichi Nozaki
Paul L. Nunez
Duane Nykamp
Matthew Oates
M. Kerry O'Banion
Raimund Ober
Gabriel Ocker
Lauren O'Connell
Cian O'Donnell
Amy Odum
Thomas Oertner
Layla Oesper
Charles Ofria
Uwe Ohler
Hisashi Ohtsuki
Mariko Okada-Hatakeyama
Kazuo Okanoya
Christopher Oldfield
Timothy O'Leary
Tanaseichuk Olga
Baldo Oliva
Bruno Olshausen
Megan O'Mara

Mary O'Neill
Arno Onken
Cees Oomens
Nikolaas Oosterhof
Lance Optican
Gergo Orban
Jean-Jacques Orban De Xivry
Christine Orengo
Amy Orsborn
Mario Orsi
Pedro Ortega
Vanessa Ortiz
James Osborne
Roman Osman
Daniel Osorio
George Oster
Andrei Osterman
Srdjan Ostojic
Justin O'Sullivan
Hans Othmer
Karen Ottemann
Thomas Otto
Thomas Ouldridge
Zhengqing Ouyang
Markus Owen
Ellis Owusu-Dabo
Diego Oyarzún
Banu Ozkan
Alberto Paccanaro
Jorge Pacheco
Marius Pachitariu
Emanuele Paci
Adam Packer
Giuseppe Paglia
Andrea Pagnani
Anand Pai
Joris Paijmans
Tiago Paixão
Patricia Palagi
Guenther Palm
Stephanie Palmer
Stefano Palminteri
Albert Pan
Tsorng-Whay Pan
Anna R Panchenko
Vijay Pande
Gaurav Pandey
Aridaman Pandit
Stefano Panzeri
Anne Paoletti
Daniela Paolotti
Elena Papaleo
Garegin Papoian
Balázs Papp
Francesco Pappalardo
Rohit Pappu
Memming Park
Andrew Parker
Joseph Parker
John Parkinson
Lucas Parra
Bogdan Pasaniuc
Richard Pastor
Kaustubh Patil
Kiran Patil
Rob Patro
Sinu Paul
Jonas Paulsen
Johan Paulsson
Paul Pavlidis
Georgios Pavlopoulos
Joshua Payne
John Pearson
Jean Peccoud
Morten Pedersen
Juan Manuel Pedraza
Christine Pedroarena
Stephan Peischl
Serge Pelet
Jorge Peña
Thomas Pence
Jian Peng
Pleuni Pennings
William Penny
David Perahia
Matjaz Perc
Josue Perez-Santiago
Rodrigo Perin
Matthew Perisin
Vipul Periwal
Alex Perkins
Andrea Perna
Nicolas Perony
Nicola Perra
Marc Perry
Arkady Pertsov
Fernando Peruani
Isabelle Peter
Christian Petersen
Dusan Petric
Linda Petzold
Giovanni Pezzulo
Jim Pfaendtner
Nico Pfeifer
Michael Pfeiffer
Benjamin Pfeuty
Andrew Philippides
Andrew Phillips
Stefano Piana
Sergei Pilyugin
Heather Pinkett
Gordon Pipa
Roger Pique-Regi
Efstratios Pistikopoulos
Igor Pivkin
Francisco Planes
Nuria Plattner
Dietmar Plenz
Joshua Plotkin
Steven Plotkin
Jagdeep Podichetty
Taras Pogorelov
Michael Poidinger
Panayiota Poirazi
Timothée Poisot
Daniel Polani
Rafael Polania
Régis Pomès
Silvina Ponce Dawson
Adam Ponzi
Art Poon
Aleksander Popel
David Posson
Mark Potse
Babak Pourbohloul
Gibin Powathil
Simon Powers
Juan Poyatos
Viola Priesemann
Boris Prilutsky
Jose Principe
U. Deva Priyakumar
Mikhail Prokopenko
Paolo Provero

Thorsten Prustel
Sergey Prykhozhij
Nataša Pržulj
Teresa Przytycka
Jose Puglisi
Bali Pulendran
Sara Pulit
Sunil Puria
Rituraj Purohit
Dale Purves
Hong Qian
Weiliang Qiu
Aaron Quinlan
Gerald Quon
Yitzhak Rabin
Mikhail Rabinovich
Nicole Radde
Predrag Radivojac
Joachim Rädler
Mark Ragan
Dobromir Rahnev
Francesco Raimondi
Paul Rainey
Glen Rains
Peter Ralph
E. Prabhu Raman
Kasper Rand
Padmini Rangamani
Aaditya Rangan
James Rankin
Benjamin Raphael
Garvesh Raskutti
Daniel Rasmussen
Randall Rasmusson
Oliver Ratmann
Magnus Rattray
Tobias Rausch
Antonio Rausell
Erzsebet Ravasz Regan
David Ray
J. Christian Ray
Monika Ray
Elizabeth Read
Jenny Read
David Reby
Maurizio Recanatini
Mario Recker
Sy Redding
Sai Reddy
Jennifer Reed
Gregory Reeves
Lynne Regan
Lars Reichl
Frank Reimann
Robert Reiner
Han Remaut
Michiel Remme
Jinsong Ren
Alfonso Renart
Jyothi Rengarajan
Lars Renner
Elisabeth Rens
Stefan A. Rensing
Leslie Reperant
Olivier Restif
Craig Reynolds
John Reynolds
Noah Ribeck
Ruy Ribeiro
Sidarta Ribeiro
John Rice
Magnus Richardson
William Richardson
Tim Ricken
Bernd Rieger
Lionel Rigoux
Todd Riley
John Rinzel
Herre Jelger Risselada
Graham Ritchie
Christian Ritz
Caitlin Rivers
Martin Robert
Elijah Roberts
David Robertson
Mark Robinson
Peter Robinson
Mattia Rocco
Miguel Rocha
Benjamin Roche
Ilia Rochlin
Ignacio Rodriguez-Brenes
Adrienne Roeder
Bjorn Rogell
Jim Rogers
Adrian Roitberg
Gianfranco Romanazzi
Sandro Romani
Natalie Romanov
Angelo Rosa
Gail Rosen
Saharon Rosset
Arnd Roth
Fritz Roth
Volker Roth
Stefan Rotter
Julien Roux
Igor Rouzine
Maga Rowicka
Alex Roxin
Deodutta Roy
Sushmita Roy
Swarup Roy
Christopher Rozell
Andrii Rozhok
Jianhua Ruan
Leonid Rubchinsky
Dan Rubenstein
Don Rubin
Jonathan Rubin
Joshua Rubin
Mikail Rubinov
Pau Rué
Adam Runions
Peter Ruoff
Eytan Ruppin
Hannes Rusch
Hans-Joachim Ruscheweyh
Raffaele Russo
Antia Rustom
Edward Ruthazer
Guy Rutter
Gustaf Rydell
Arni S.R. Srinivasa Rao
Lao Saal
Karen Sachs
Frank Sachse
Lawren Sack
Roberto Saenz
Erik Sahai
Maneesh Sahani
Yuichi Sakumura
Marco Salemi
Andrej Sali

Howard Salis
Richard Sallari
Xavier Salvatella
Steven Salzberg
Inés Samengo
Jason Samonds
Adam Sanborn
Alvaro Sanchez
Chris Sander
Yves-Henri Sanejouand
Sriram Sankararaman
Michel Sanner
Fidel Santamaria
Francisco Santos
Mauro Santos
Casim Sarkar
Sridevi Sarma
Tatsuya Sasaki
Rahul Satija
Rahul Satija
Tamaki Sato
Ausra Saudargiene
Herbert Sauro
Olivier Saut
Thomas Sauter
Francesco Savelli
Gregory Sawicki
Nathaniel Sawtell
Samuel Scarpino
William Schafer
Lars Schäfer
Ralf Schäfer
Anna Schapiro
Birgit Scharf
Robert Scharpf
Michael Schatz
Sascha Schäuble
Robert Scheidt
Nancy Schellhorn
Jennifer Schellinck
Harald Scherm
Richard Scheuermann
Volker Scheuss
Kaspar Schindler
Tamar Schlick
Patrick Schloss
Michael Schmuker
Reinhard Schneider
Elad Schneidman
Michael Schnieders
Jan Schnupp
Geoffrey Schoenbaum
Benjamin Scholl
Jacqueline Scholl
Torsten Schöneberg
Gideon Schreiber
Jessica Schrouff
Ora Schueler-Furman
Alexander Schug
Klaus Schulten
André Schultz
Wolfram Schultz
Linus Schumacher
David Schwab
Daniel Schwartz
Russell Schwartz
Ulrich Schwarz
Torsten Schwede
Nicolas Schweighofer
Jason Schweinsberg
Lars Ole Schwen
Joost Schymkowitz
Philippe Schyns
Annalisa Scimemi
Jacob Scott
Matthew Scott
Timothy Secomb
Daniel Seeliger
Rebecca Segal
Ayellet Segrè
Daniel Segrè
Luc Selen
Allen Selverston
Agnese Seminara
Chandan Sen
Taner Sen
Ryan Senger
Walter Senn
Luis Serrano
October Sessions
Manu Setty
Ugur Sezerman
Pedro Sfriso
Ioannis Sgouralis
Nigam Shah
Premal Shah
Babak Shahbaba
Vahid Shahrezaei
Leili Shahriyari
Yuk Sham
Maoz Shamir
Ladan Shams
A.S. Shan
Yibing Shan
Mukherji Shankar
Daryl Shanley
Robert Shapley
Tatyana Sharpee
Thomas Sharpton
Hagit Shatkay
Nir Shavit
Michael W. Shaw
Amarda Shehu
Jana Shen
Ronglai Shen
Xilin Shen
Woodrow Shew
Xinghua Shi
Darryl Shibata
Tatsuo Shibata
Denis Shields
Jay Shin
Abbas Shirinifard
Ilya Shmulevich
Brian Shoichet
Indira Shrivastava
Jianwei Shuai
Bailu Si
Sachdev Sidhu
Kyriaki Sidiropoulou
Heike Siebert
Ivo Siekmann
Fabian Sievers
Mariano Sigman
Mary Silber
Jerson Silva
Jonathan Silva
Petr Simecek
Scott Simon
Kai Simons
Jacob Simonsen
Lone Simonsen
Jeremy Simpson
Abhyudai Singh

Mona Singh
Saurabh Sinha
Fabian Sinz
Mark Sisterson
Frances Skinner
Jane Skok
Marcin Skwark
Tony Slaba
Boris Slepchenko
Timo Smieszek
Gavin Smith
Gordon Smith
Jeremy Smith
Matthew Smith
Matthew Smith
Maurice Smith
V. Anne Smith
Gordon Smyth
Michael Sneddon
Kim Sneppen
James Sneyd
Chris Snow
Joel Snyder
Eric Sobie
Joshua Socolar
Johannes Söding
Johannes Soeding
Christian Soeller
Mohammad Soheilypour
Artem Sokolov
Igor Sokolov
Soroosh Solhjoo
Trygve Solstad
Fritz Sommer
Marc Sommer
Hyun-Seob Song
Jimin Song
Sen Song
Wenjun Song
Swapnil Sonkusare
Nikolaus Sonnenschein
Richelle Sopko
Tobin Sosnick
Roberto Sotero Diaz
Stamatios Sotiropoulos
Marcos Sotomayor
Samuel Soubeyrand
Vassili Soumelis
Christian Specht
Paul Spellman
Fabian Spill
Michael Spratling
Henning Sprekeler
Michael Springer
Francesca Spyrakis
Mandyam Srinivasan
Ganesh Sriram
Peter Stadler
Tanja Stadler
Scott Stagg
Alexandros Stamatakis
George Stan
Daron Standley
Kenneth Stanley
Phillip Stansfeld
Joshua Starmer
Stephen (Steve) Stearns
Steven Steinway
Federico Stella
Ulrich Stelzl
Olaf Stemmann
Martin Stemmler
Greg Stephens
Michael Stern
Dagmar Sternad
Ian Stevenson
Neil Stewart
Heinrich Sticht
Ger Stienen
Gerhard Stock
Alan Stocker
Thomas Stockner
John Stone
Katherine Storrs
Ariana Strandburg-Peshkin
Ronny Straube
Andrew Straw
Sergei Strelkov
Thomas Stricker
Roland Strong
Cory Strope
Marc Strous
David Strutt
Romain Studer
Wolfgang Stuerzl
Michael Stumpf
Andreas Stumpner
Mark Styczynski
Andrew Su
Yukiko Sugi
Sergei Sukharev
Pavel Sumazin
Mark Sun
Yidi Sun
Myong-Hee Sung
Vikram Sunkara
Shamil Sunyaev
Philip Supply
Ivan Surovtsev
Shinsuke Suzuki
Nicholas Swindale
Gyorgy Szabo
Ewa Szczurek
Oliwia Maria Szklarczyk
Josué Sznitman
Joel Tabak
Enzo Tagliazucchi
Shoji Takada
Daniel Takahashi
Cheemeng Tan
Kai Tan
Kean Ming Tan
Xiaodong Tan
Mark Tanaka
Shigeru Tanaka
Chao Tang
Jing Tang
Min Tang
Yinjie Tang
Zhengzheng Tang
Michael Tangermann
Bertrand Tanner
Sonia Tarazona
Adi Tarca
Morgan Taschuk
Peter Tass
Nicholas Tatonetti
Cormac T. Taylor
Jordan Taylor
Peter Taylor
Tracy Teal
Kirsten Ten Tusscher
Pieter Rein Ten Wolde
Hiroki Terashima

Andrew Teschendorff
Christian Tetzlaff
Tom Tetzlaff
Bas Teusink
Shivendra Tewari
Johannes Textor
Sharma Thankachan
Amantha Thathiah
Mukund Thattai
Lucas Theis
Raghuram Thiagarajan
Denis Thieffry
Bertrand Thirion
Dave Thirumalai
Peter Thomas
Kevin Thornton
Simon Thorpe
Kevin Thurley
Xiao-Jun Tian
D. Peter Tieleman
Paul Tiesinga
Michael Tildesley
Nicholas Timme
Winston Timp
Marcus Tindall
Abhinav Tiwari
Michele Tizzoni
Dror Tobi
Michal Toborek
Michael Tolstorukov
Iuliana Toma-Dasu
Michael Tomasello
Cristian Tomasetti
Peter Tompa
Weida Tong
Sina Tootoonian
Chad Topaz
Maya Topf
Adriano Tort
Silvio Tosatto
Ágnes Tóth-Petróczy
Jonathan Touboul
Nicolas Touret
Jacques-Donald Tournier
Taro Toyoizumi
Jan Traas
Zlatko Trajanoski
Anna Tramontano
Roger Traub
Rui Travasso
Werner Treptow
Alessandro Treves
Victor Trevino
Jochen Triesch
Iñaki Troconiz
Carl Troein
Wilson Truccolo
George Tseng
Konstantinos Tsetsos
Lev Tsimring
Misha Tsodyks
Lisa Tucker-Kellogg
Catalina Tudor
Pierre Tuffery
Tamir Tuller
Ignacio Tunon
John Turchi
Marc Turcotte
Denes Turei
Nicholas Turk-Browne
Erkan Tuzel
John Tyson
Jung-Ying Tzeng
Duygu Ucar
Hiroki Ueda
Frank Uhlmann
Nachum Ulanovsky
Alfred Ultsch
David Umulis
Ron Unger
Swapna Uplekar
Brigita Urbanc
John Ussher
Naveen Vaidya
Sandor Vajda
Ady Vaknin
Ilya Vakser
Alfonso Valencia
Robert Van Beers
Jeremy Van Cleve
Johan Van De Koppel
Albert Van Den Berg
Wouter Van Den Bos
Herman Van Der Kooij
Roemer Van Der Meij
David Van Der Spoel
Jacques Van Helden
Vera Van Noort
Hans Van Oosterwyck
Mark Van Rossum
Rob Van Spanning
Jeroen Van Zon
Vincent Vanburen
Benjamin Vandersluis
Fabio Vandin
Marco Vanoni
Marta Varela
Jeffrey Varner
Nelle Varoquaux
Charles Vaske
Dimitrios Vavylonis
Catia Vaz
Shruthi Vembar
Michele Vendruscolo
Venessa Venturi
Gennady Verkhivker
Deeptak Verma
Julius Verrel
Karin Verspoor
Jean-Philippe Vert
Allegra Via
Cecile Viboud
Neil Vickers
Tamás Vicsek
Mathukumalli Vidyasagar
Matthieu Vignes
Mauno Vihinen
Jose Vilar
Alessandra Villa
Nathalie Villa-Vialaneix
Emmanuel Villermaux
Benjamin Vincent
Martin Vinck
Mark Viney
Martin Vingron
P.K. Vinod
Olga Vitek
Michel Vivaudou
Kristian Vlahovicek
Yoram Vodovotz
Vincent Voelz
Joshua Vogelstein
Raphael Voituriez
Margaritis Voliotis

Vladislav Volman
Erik Volz
Tobias Von Der Haar
Gregory Voth
Wim Vranken
Gert Vriend
Patrik Vuilleumier
Edward Vul
Krzysztof Wabnik
Thomas Wachtler
Alex Wade
Rebecca Wade
Jacques Wadiche
Wytse Wadman
Eric-Jan Wagenmakers
Tor Wager
Hermann Wagner
Lindi Wahl
S. Wahl
Yuichi Wakamoto
Aleksandra Walczak
Jerome Waldispühl
Lourens Waldorp
Levi Waldron
Jacco Wallinga
Mark Walton
Jianmin Wan
Matthew Wanat
Brian Wandell
Chen Wang
Jin Wang
Junwen Wang
Minghui Wang
Pei Wang
Qingyun Wang
Rachel Wang
Rui Wang
Wei Wang
Wenyi Wang
Xiao Wang
Xiaowo Wang
Xuefeng Wang
Zhihui Wang
Melissa Ward
Joanna Wares
Tandy Warnow
Silvio Waschina
Mark Wass
Tsjerk Wassenaar
Wyeth Wasserman
Karen Watanabe
Dean Waters
Michael Watson
Jonathan Wattis
Greg Wayne
Helen Wearing
Barbara Webb
Colleen Webb
Caleb Webber
Matti Weckström
Michael Wehr
Chih-Hsuan Wei
Guowei Wei
Kunlin Wei
Martin Weigt
Cornelis Weijer
Seth Weinberg
Ariel Weinberger
Leor Weinberger
Alan Weinstein
Ariel Weinstein
John Weisel
Franz Weissing
Joshua Weitz
Brian Weitzner
David Welch
John Welch
Lonnie Welch
Wolfgang Wenzel
Justin Werfel
Benjamin Werner
Joel Wertheim
Ralf Wessel
John Westbrook
Pål Westermark
Bruce Wheeler
Nicole Wheeler
Lisa White
Paul Whitford
Steven Whitten
David Whitworth
Lutz Wiegrebe
Sadie Wignall
Geoff Wild
Brian Wilhelm
Thomas Wilhelm
Claus Wilke
Amy Williams
C. David Williams
David Williams
John Williams
Robert Williams
Ben Willmore
Hugh Wilson
Robert Wilson
Ned Wingreen
Christof Winter
Thomas Wischgoll
David Wishart
Christoph Witzel
Ulrich Witzel
Shoshana Wodak
Dominik Wodarz
Florentin Woergoetter
Guy Wolf
Charles Wolgemuth
Aaron Wong
Chung Wong
Dan Woodcock
Henry Woodcock
Ben Woodcroft
Robert Woods
Mark Woolhouse
Willy Wriggers
Chunlei Wu
Di Wu
Yilin Wu
Stefan Wuchty
Andreas Wutz
Joao Xavier
Jinxiang Xi
Xiao Xiao
Lu Xie
Xiaohui Xie
Jianhua Xing
Momiao Xiong
Heng Xu
Huafeng Xu
Lin Xu
Xiaojun Xu
Chuan Xue
Yu Xue
Gur Yaari
Yoshihiro Yamanishi

Dan Yamin
Daniel Yamins
An-Suei Yang
Ence Yang
Lee-Wei Yang
Lun Yang
Wan Yang
Wei Yang
Xiaojing Yang
Xinan Yang
Yufeng Yang
Thomas Yankeelov
Chen Yanover
Yosi Yarom
Vladimir Yarov-Yarovoy
Andrew Yates
Christopher Yeo
Carl Yeoman
Ka Yee Yeung
Pierre Yger
Kevin Yuk-Lap Yip
Hiroki Yokota
Nir Yosef
Lingchong You
Alistair Young
Jamey Young
Peter Young
Samuel Young
Haiyuan Yu
Ji Yu
Jin Yu
Yinyin Yuan
Bojan Zagrovic
Andrew Zalesky
Peter Zandstra
Bas-Jan Zandt
Jurgen Zanghellini
David Zanuy
Arieh Zaritsky
Kourosh Zarringhalam
Jeremiah Zartman
Christoph Zechner
Jochen Zeil
Alexander Zelikovsky
Jonathan Zelner
Yuval Zelnik
Friedemann Zenke
Shuhei Zenno
Helen Zgurskaya
Xiaowei Zhan
Bin Zhang
Jianzhi Zhang
Le Zhang
Shihua Zhang
Shu-Dong Zhang
Sulin Zhang
Ying Zhang
Zhaolei Zhang
Jichao Zhao
Liping Zhao
Jie Zheng
Qiping Zheng
Wenjun Zheng
Xiaobin Zheng
Shan Zhong
Changsong Zhou
Jian Zhou
Joseph Zhou
Cheng Zhu
Fangqiang Zhu
Jiang Zhu
Jie Zhu
Jun Zhu
Luoding Zhu
Shanfeng Zhu
Igor Zhulin
Sasha Zill
Michal Zochowski
Aldert Zomer
Ali Zomorrodi
Justin Zook
Jinfeng Zou
Daniel Zuckerman
Anze Zupanic
